# Trauma is a global issue

**DOI:** 10.3402/ejpt.v4i0.20419

**Published:** 2013-03-04

**Authors:** Ulrich Schnyder

**Affiliations:** Department of Psychiatry and Psychotherapy, University Hospital, CH-8091 Zurich, Switzerland

Trauma is a global issue. To give just one example, terrorism is a phenomenon that can only be understood and dealt with constructively by adopting a cross-national, cross-cultural perspective. I feel very strongly that the field of traumatic stress research should develop collaborations, and ultimately structures as well, that enable us to optimally respond to those tasks that are best addressed by means of international collaboration.

Also, trauma is more than just “psychological trauma”. I believe trauma can best be understood using an interdisciplinary, multiprofessional, biopsychosocial approach. Therefore, we should aim at diversifying our professional dialogue by actively reaching out to related disciplines. To better understand trauma, we have to study its devastating effects, but learn more about resilience to stress as well. I also think trauma work should be integrated in the mainstream of psychology and medicine, including psychiatry and public health, as well as in neuroscience, sociology, anthropology, law, and many other fields. Thus, we should make an even greater effort to welcome professionals from these disciplines to share with us their knowledge and expertise.

During my term as president of International Society for Traumatic Stress Studies (ISTSS), the Board of Directors created a new strategic plan. In April 2010, at the ISTSS mid-year Board meeting in Zurich, we developed a set of global values and visions. We recognized that traumatic stress is a global issue, that we seek to have a stronger global impact on trauma-related issues, and that we could speak with a stronger voice, if we represented larger numbers of trauma professionals around the world. We committed to value worldwide collaboration over competition and to try to ensure that the needs of all nations are met. Finally, we agreed we feel responsible to attend for those who are without local trauma support.

The Board of Directors voted this strategic goal as a high ISTSS priority. As a result, the “Global Initiative” was created and a project team was charged to invite broad input from key stakeholders to discover stakeholder views, priorities, and preferences; learn about current best practices in global relationships, what others are successfully doing and what is possible, in order to make as informed a recommendation as possible; make recommendations for alternative business models to consider; and, finally, in consultation with stakeholders, examine these alternative models and build consensus around proposals that can be supported by all.

The project team, consisting of representatives from ISTSS and the majority of its affiliate societies, first tried to identify various options for a new organizational structure of ISTSS. One model we considered was a global collaboration of organizations worldwide with an interest in advancing the field of traumatic stress, with a confederation structure that would include the ISTSS, its current affiliates, plus potentially other associations as well. A second model was the “Global Society for Traumatic Stress Studies”, a new umbrella organization, that is, a federation much along the lines of how the ESTSS is currently structured. A third model was the creation of a North American section or affiliate of the (otherwise unchanged) ISTSS—a section that would meet the needs of the US-based constituency of the ISTSS. While the ISTSS Board of Directors strongly encouraged the project team to further develop the various options, other stakeholders were much less enthusiastic about changing the organizational structure of ISTSS.

Therefore, we adapted our strategy and started thinking more pragmatically, asking ourselves what we might actually be doing if one of the envisioned structural models were fully implemented. Keeping in mind the initial purpose of the Global Initiative (greater global impact, greater peer-to-peer balance among societies, addressing US-specific needs), and applying the principle of “form follows function”, the project team developed the following three concrete action packages:

- No-cost membership: A new membership category for ISTSS, offering a restricted range of benefits, to meet the basic communal needs of isolated, low-income professionals worldwide, while also offering a valuable connection for more affluent professionals who have their primary memberships in other organizations. No-cost membership would increase the impact of programs and services provided by ISTSS and partnering organizations by engaging with a larger community of professionals who have interest in traumatic stress. “Affiliated” no-cost ISTSS membership would apply to dues-paying members of affiliated organizations. “Corresponding” no-cost ISTSS membership would apply to those with no paid membership in either ISTSS or an affiliated organization. Details and implementation of these new membership options must involve consultation and coordination with affiliated organizations.
- ISTSS meetings outside North America: One-day educational meetings would be held around the world, in collaboration with local societies when feasible. In addition, larger ISTSS regional conferences on traumatic stress would be offered in places where no strong STSS representation exists. This type of meeting would be used to facilitate the formation of new traumatic stress organizations.
- Global collaboration: The idea of this action package was to convene organizations interested in traumatic stress and to work alongside each other on an equal basis. Participants would identify objectives, facilitate development, and coordinate activities of global importance. Organizations would be free to determine whether or not to be involved in particular initiatives. This effort would begin with ISTSS and its affiliate societies, but it is intended to encompass the broader trauma community in the future.

The three action packages were approved, with minor amendments, by the Board of ISTSS in May 2012. On October 29, 2012, on the occasion of its annual meeting in Los Angeles, the ISTSS hosted the first one-day meeting of the Global Collaboration. Representatives from ISTSS and its affiliate societies engaged in a very lively and inspiring discussion. In a historic moment, the group achieved agreement to work collaboratively, focusing on one global issue to start with, namely childhood abuse and neglect and the latent impact of that abuse. Child abuse and neglect is clearly a global public health problem that requires a global solution. The Global Collaboration decided to collect guidelines from around the world that would provide the basis for a synthesized core guideline for prevention and treatment that can be customized for specific cultural contexts. The guideline will primarily be aimed at professionals. In addition, the Collaboration will compile an information guide aimed at those affected by childhood abuse and neglect. This will raise awareness of the issue, improve the way individuals of all ages who are affected by childhood abuse and neglect are detected, supported, assessed and treated, leading to significant improvements in health and wellbeing. Capitalizing on the latest developments in technology, the Collaboration aims to disseminate these guidelines using an application for mobile electronic devices that will allow for worldwide distribution and cultural customization. The Collaboration is currently working together to develop a proposal to secure funding to develop the guidelines and the application. Furthermore, participants agreed to discuss with their societies and boards the various ways they can and will join ISTSS in contributing to this effort.

The Global Collaboration is chaired by ISTSS vice-president Miranda Olff for the time being. The next face-to-face meeting will take place during the 13th ESTSS conference in Bologna in June 2013. I am truly delighted and grateful to see the Global Initiative coming to fruition. Trauma is a global issue. I wish the Global Collaboration all the success it deserves!

*Ulrich Schnyder* Past President, European Society for Traumatic Stress Studies ESTSS Past President, International Society for Traumatic Stress Studies Professor of Psychiatry and Head, Department of Psychiatry and Psychotherapy University Hospital CH-8091 Zurich, Switzerland Email: ulrich.schnyder@access.uzh.ch

